# Biological serial block face scanning electron microscopy at improved z-resolution based on Monte Carlo model

**DOI:** 10.1038/s41598-018-31231-w

**Published:** 2018-08-28

**Authors:** Q. He, M. Hsueh, G. Zhang, D. C. Joy, R. D. Leapman

**Affiliations:** 1 National Institute of Biomedical Imaging and Bioengineering, NIH, Bethesda, MD 20892 USA; 2 Gatan, Inc., 5794W. Las Positas Blvd, Pleasanton, CA 94588 USA; 3 Department of Material Science and Engineering, University of Tennessee, Knoxville, TN 37996 USA; 4 Center for Nanophase Material Sciences, Oak Ridge National Laboratory, Oak Ridge, TN 37831 USA

## Abstract

Serial block-face electron microscopy (SBEM) provides nanoscale 3D ultrastructure of embedded and stained cells and tissues in volumes of up to 10^7^ µm^3^. In SBEM, electrons with 1–3 keV energies are incident on a specimen block, from which backscattered electron (BSE) images are collected with *x*, *y* resolution of 5–10 nm in the block-face plane, and successive layers are removed by an *in situ* ultramicrotome. Spatial resolution along the *z-*direction, however, is limited to around 25 nm by the minimum cutting thickness. To improve the *z-*resolution, we have extracted depth information from BSE images acquired at dual primary beam energies, using Monte Carlo simulations of electron scattering. The relationship between depth of stain and ratio of dual-energy BSE intensities enables us to determine 3D structure with a ×2 improvement in *z-*resolution. We demonstrate the technique by sub-slice imaging of hepatocyte membranes in liver tissue.

## Introduction

Over the past decade, techniques have been developed, which enable nanoscale 3D imaging of large biological tissue volumes^1–10^. In these approaches, tissues prepared by fixation and staining are embedded in plastic to provide specimen blocks consisting of heavy-atoms bound to cellular structures contained in a light-atom polymer matrix^11,12^. A scanning electron microscope (SEM) is then used to image the surface of specimen block either in conjunction with (1) a built-in ultramicrotome, which cuts off successive layers in a technique known as serial block-face electron microscopy (SBEM) – otherwise referred to as serial block-face scanning electron microscopy (SBF-SEM)^1–4,9,10,13–15^, or in conjunction with (2) a focused ion beam (FIB), which sputters successive layers from the surface of the block face in the technique known as FIB-SEM^5–10,16–19^. These capabilities for imaging large volumes on the scale of a few nanometers provide an anatomical perspective of tissues and their constituent cells, which is not available from conventional transmission electron microscopy^15–17^. Each approach enables us to obtain quantitative models of tissue architecture and thereby to generate new hypotheses that relate cellular anatomy to biological function. Much of the impetus for developing the methods has come from interest in applying them to dense mapping of neuronal circuitry in the nervous system^1–4,16,17,20,21^. Both techniques image the sample block face using a backscattered electron (BSE) detector to collect the signal from heavy atoms that stain cellular structures, since the elastic backscattered yield has a strong dependence on atomic number. In SBEM, the specimen height is raised by some set amount (typically in the range 25–100 nm) following acquisition of each BSE image, after which the block is cut by the ultramicrotome to expose a new flat surface. Through repetition of this process, it is possible to acquire a stack of BSE block-face images, which resemble TEM images of conventionally stained thin sections, after reversal of the BSE contrast.

Both the FIB-SEM and SBEM techniques enable imaging of large sample volumes with a pixel size of about 5 nm in the *x*-*y* plane of the block face^13,14,18,19^. FIB milling is a relatively slow process, which limits the total volume that can be analyzed, typically, to less than 10^5^ µm^3^. However, the cutting speed is much faster in SBEM, thus enabling acquisition of volumes as large as 10^7^ µm^3^ ^1–4^. In FIB-SEM the spatial resolution of ~5 nm is approximately isotropic in *x*, *y* and *z* since the focused ion beam can erode arbitrarily thin surface layers^10^. However, since the minimum slice thickness cut by the microtome is approximately 25 nm, conventional SBEM has poorer spatial resolution in the direction perpendicular to the block face (*z*-direction)^1–4,13–15^.

**Figure 1:**
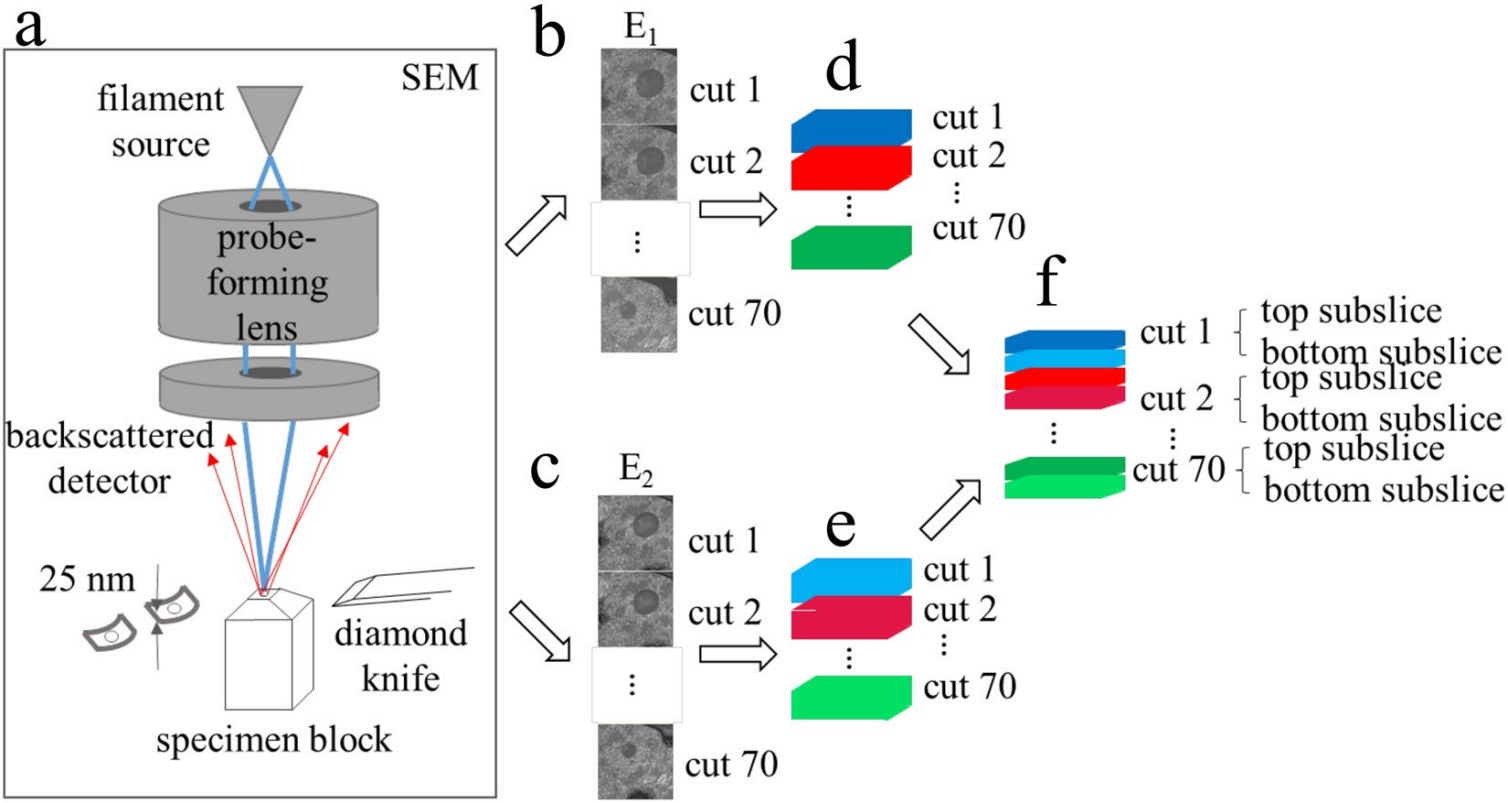
Diagram of sub-slice imaging in the SBEM. (**a**) Surface of the sample block is imaged by scanning a focused electron probe from a field emission source across a region of interest and collecting backscattered electrons (BSEs) with an annular detector. Sample block is raised by height ≥ 25 nm, and a section of that thickness is shaved off using a diamond knife mounted in the SBEM’s *in situ* microtome. The newly exposed surface is then re-imaged. This process is repeated until an image stack is collected from the desired sample volume, 70 slices in this illustration. **(b**,**c)** Series of backscattered electron images captured at energy *E*_1_ and *E*_2_, respectively. **(d**,**e)** 3D reconstruction of single-energy image series from **(b**,**c)**, respectively. **(f)** Dual-energy reconstruction gives sub-slice resolution.

The high throughput technique of SBEM, illustrated schematically in Fig. 1a, shows the arrangement of the microtome, sample block and backscattered electron (BSE) detector. The signal from the BSE detector provides strong contrast from heavy atoms of stain situated near the surface of the specimen block, as indicated by the set of backscattered images collected after each cut (Fig. 1b,d). Here, we explore the feasibility of improving the *z*-resolution in SBEM by acquiring backscattered electron images with different primary energies (Fig. 1c,e) so that the incident electron probe is concentrated at different subsurface depths, which provides subslice resolution (Fig. 1f). This idea has been described by Boughorbel *et al*.^22^ and by de Goede *et al*.^23^, who developed a technique (ThruSight) for FEI/Thermo Fisher Scientific SEMs^24^, based on a blind deconvolution method that iteratively fits a series of images recorded at different primary beam energies to deduce the most likely 3D subsurface structure. Both the approach that we consider here and the blind deconvolution approach are based on the property that backscattered images generated at different electron primary beam energies from plastic-embedded stained biological structures exhibit high linearity with respect to stain density, which enables models of image formation based on linear convolutions. Since the penetration depths are dependent on the primary beam energies, multiple images acquired at increasing beam energies contain highly overlapping depth information. In principle, the blind deconvolution can be performed knowing neither the depth distribution of the stain, nor the point spread function (PSF). This PSF reflects the interactions between the electron beam and heavy atoms located at different depths within a light atom matrix, which results in different backscattered electron signals.

However, the blind deconvolution method does not consider a physical model of electron scattering. Instead, both the depth distributions of the stain and the PSF are determined using an iterative procedure incorporating prior knowledge or constraints such as non-negativity. The blind deconvolution approach is known to constitute a challenging non-convex optimization problem with the potential for non-unique solutions. In contrast, our approach derives a discretized PSF directly using Monte Carlo simulations based on electron scattering cross sections for electron probes of different energies interacting with a specimen of known composition. Derivation of the PSF enables a more direct inverse computation of the stain distribution from the backscattered images, and allows us to test the assumption of linearity of signals for a known concentration of stain that matches the measured stain concentration in our specimens. Thus, we believe that our model-based approach and blind deconvolution approach are complementary. Our present aim is to obtain a complementary physically based model incorporating electron scattering theory through a Monte Carlo simulation, which enables us to reconstruct subsurface features, to estimate errors in the reconstruction, and to determine the fundamental limits of subsurface imaging that are imposed by radiation damage, which results in collapse of the specimen under electron irradiation.

In earlier work, Hennig and Denk used Monte Carlo simulations to model the BSE signal from plastic-embedded heavy metal stained biological specimens^25^. These authors found that the image contrast is linear for typical stain densities, and that the interaction volume is concentrated along the incident probe direction. This linearity of the generated BSE signal as a function of heavy atom concentration is important when one tries to extract 3D information within subsurface layers of the specimen block, which is mainly composed of the embedding material consisting of light atoms with a minor component of heavy atoms. The Monte Carlo simulations show quantitatively how the BSE signal is generated within the electron interaction volume. We have determined experimentally (see Methods) that the heavy atom content of the stained liver sample used in the present work is ~1 atomic percent, with a maximum local concentration of ~3 atomic percent. Our Monte Carlo simulations show that the BSE signal is linear in this concentration range with a maximum value of 5.8% of the incident probe fluence for a landing energy of 1.0 keV; significant departures from linearity only occur at stain concentrations above 10 atomic percent (Supplementary Fig. 5). Incident electrons of higher energy have a greater range inside the specimen block than electrons of lower energy since they travel further before inelastic scattering mechanisms reduce the energies of the electrons to the extent that they can no longer escape from the surface and contribute to the BSE signal. The penetration depth is mainly determined by inelastic scattering because the total inelastic scattering cross section has a relatively weak dependence on atomic number, so that a minor component of heavy atoms does not make a large contribution to the penetration depth^26^. Moreover, since inelastic scattering events are associated with small scattering angles, the interaction volume is strongly localized in the forward direction of the electron probe, i.e., along the *z*-axis. At low primary electron energies, only features located close to the block surface give rise to strong contrast in BSE images, whereas at higher primary electron energies, features deeper inside the block surface display high contrast as well. Thus, BSE images at different primary electron energies provide information about the depth of a nanoscale feature stained with heavy atoms.

We first derive an algebraic formulation for deducing the stain distribution as a function of depth below the block face based on BSE images acquired at multiple landing energies. Then, we perform Monte Carlo simulations^27–30^ to compute contrast from well-defined regions of stain located at different depths in a model specimen block, and hence determine the parameters required to apply an inverse computation on experimental data. Finally, we test the approach to improve the visibility of endoplasmic reticulum membranes in mouse hepatocytes.

## Results

### Determination of 3D stain distribution from electron backscattering coefficients

The electron backscattering coefficient *η* for electron landing energy *E*_*m*_ is defined by the ratio of the number of electrons per unit area *B* that backscatter from the specimen surface to the number of electrons per unit area *J*_0_ incident on the specimen surface (i.e., fluence). For a stained biological structure embedded in plastic, the backscattering coefficient *η* is a function of position *x*, *y* and landing energy *E*_*m*_, and can be written as:

1 $${\eta }(x{,}y{,}{{E}}_{{m}})=\frac{{B}(x{,}y{,}{{E}}_{{m}})}{{{J}}_{{0}}}={\int }_{z=0}^{\infty }{A}(z{,}{{E}}_{{m}}){S}(x{,}y{,}z){dz}$$

where *B*(*x*, *y*, *E*_m_) is the number of electrons per unit area backscattered from the specimen at position *x*, *y* for landing energy *E*_*m*_; *S*(*x*, *y*, z) is the 3D distribution of heavy-atom stain density in the specimen block (i.e., number of heavy atoms per unit volume); and *A*(*z*, *E*_*m*_) describes the interaction of the incident electrons of landing energy *E*_*m*_ with the plastic embedding material containing heavy atoms located at depth *z*. The quantity *A*(*z*, *E*_*m*_) has dimensions of area and can be considered as a scattering cross section. Equation 1 only includes electrons that are backscattered from the heavy atoms of the stain and excludes electrons that are backscattered from the uniform plastic embedding material, since those contribute a constant background to the backscattering coefficient, which can be readily subtracted. As described in the Monte Carlo simulations of Hennig and Denk^25^, as well as in our simulations below, the point spread function of the incident electrons is narrow in the *x*, *y* plane, so that it is possible to separate the z-dependence of the backscattered electron signal from the *x*, *y* dependence. It is then possible to discretize Equation 1 with respect to depth *z* at each *x*, *y* pixel by introducing subsurface layers of thickness *δz*:

2 $${B}(x{,}y{,}{{E}}_{{m}})=\sum _{n=1}^{N}{A}({{z}}_{{n}}{,}{{E}}_{{m}}){S}(x{,}y{,}{{z}}_{{n}})[{{J}}_{{0}}{\delta }z]$$

Equation 2 can be written in matrix notation as:

3 $${{B}}_{{m}}=[{{J}}_{{0}}{\delta }z]{{A}}_{{nm}}{{S}}_{{n}}$$

where terms on the right side of the equation are implicitly summed over the index *n*, which appears twice. In Equation 3, we have now omitted the *x*, *y* coordinates since we are able to treat the *z*-coordinate independently due to the narrow point spread function of the incident electron probe in the *x*, *y* plane. As a further simplification of Equation 3, we abbreviate *B*(*E*_*m*_) = *B*_*m*_ and *S*(*z*_*n*_) = *S*_*n*_. Then, writing **B** = *B*_*m*_ and **S** = *S*_*n*_, and considering the case for which m = 1…. *N* and n = 1…. *N*, i.e., **A** = *A*_*nm*_ is a square *N* × *N* matrix, we can express Equation 3 as:

4 $${\bf{B}}=[{{J}}_{{0}}{\delta }z]{\bf{AS}}$$

Equation 4 can then be solved for **S** by computing the inverse of **A**, i.e., the matrix **A**^−**1**^, which gives:

5 $${\bf{S}}=\frac{{{\boldsymbol{A}}}^{-1}{\boldsymbol{B}}}{[{{J}}_{{0}}{\delta }z]}$$

In practice, it is not possible to solve Equation 5 for an arbitrary number of layers using an equivalent number of landing energies because many coefficients of the matrix **A** approach zero so that the inverse matrix **A**^−**1**^ is not well defined. In this study, we consider only the case of *N* = 2, i.e., a 2 × 2 matrix, in which two landing energies are used to determine the stain distribution in two subsurface layers. We can now write:

6 $$(\begin{array}{c}{S}_{1}\\ {S}_{2}\end{array})=[\frac{1}{{J}_{0}\delta z}]{(\begin{array}{cc}{A}_{11} & {A}_{12}\\ {A}_{21} & {A}_{22}\end{array})}^{-1}(\begin{array}{c}{B}_{1}\\ {B}_{2}\end{array})$$

where the coefficients of the matrix **A** are determined directly from the Monte Carlo simulations of lead stained (3 atomic %) cuboids located in layer 1 and layer 2 of a light atom matrix with a composition corresponding to an epoxy resin embedding material, as described below. It is convenient to use units of nanometers for all quantities in Equation 6.

The *z*-resolution in SBEM is limited by the minimum cutting increment of ~25 nm, and an ability to halve the *z*-resolution by acquiring datasets at just two primary beam energies represents a substantial advance. Here, we therefore do not consider datasets acquired using multiple primary beam energies, such as the ones acquired for analysis by the blind deconvolution method. In our experience, the signal-to-noise ratio in the image stacks decreases substantially with increasing numbers of primary beam energies since the maximum total electron fluence is limited by damage-induced shrinkage of the block.

### Monte Carlo simulations of sub-surface image contrast as a function of primary beam energy

**Figure 2:**
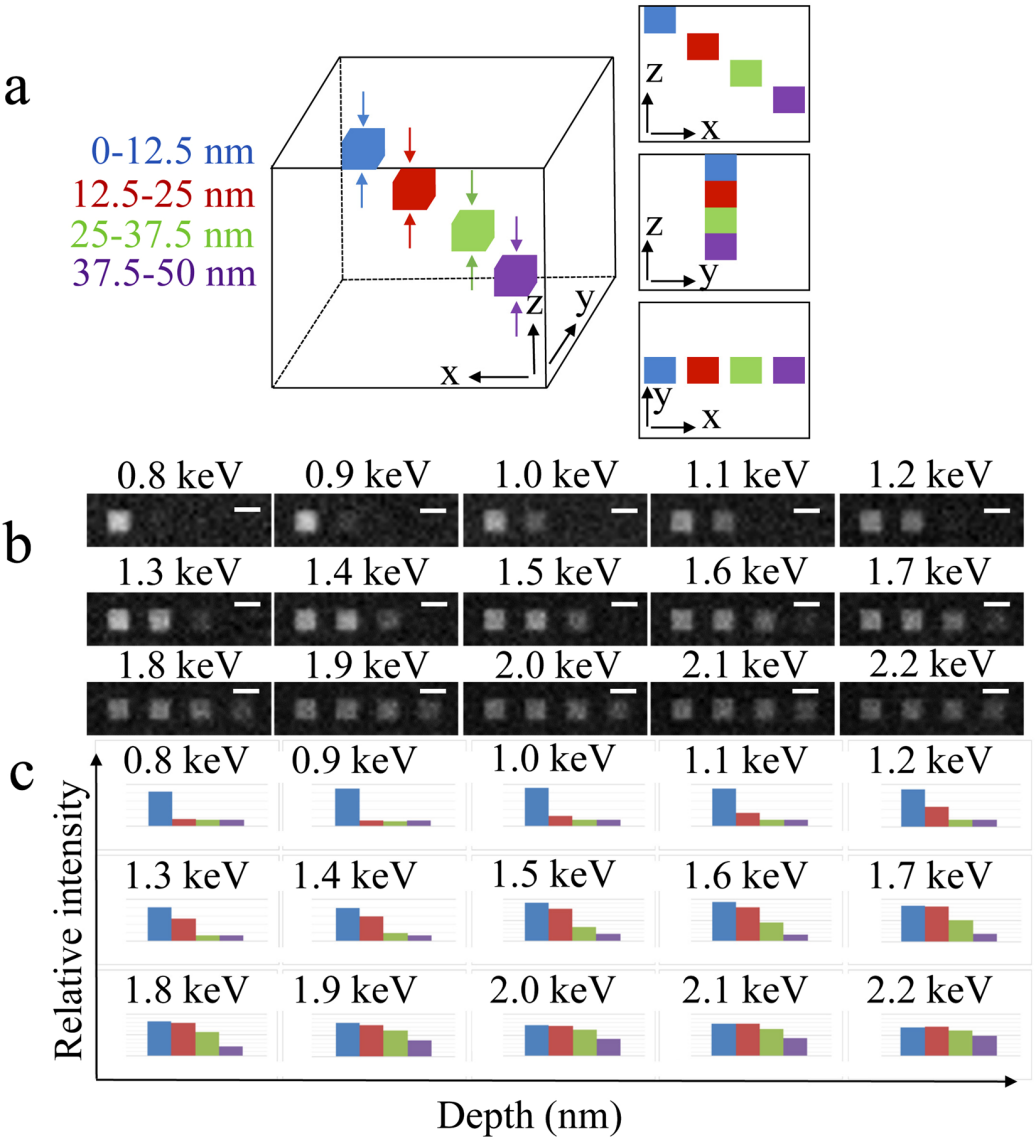
Monte Carlo simulation model and results. (**a**) Three-dimensional geometrical model for 50 nm × 50 nm × 12.5 nm cuboids stained with 3% lead located at different depths in an 800 nm × 800 nm × 800 nm epoxy block, together with views in the *x*-*z* plane, *y*-*z* plane and *x*-*y* plane. Centers of cuboids are located 6.25 nm, 18.75 nm, 31.25 nm and 43.75 nm, respectively, from the top surface of the block from left to right. Dimension in *z* is not drawn to scale. **(b)** Simulated backscattered images at primary beam energies from 0.8 keV to 2.2 keV with an interval of 0.1 keV. **(c)** Corresponding intensity profiles for cuboids shown in **(b)**. These plots provide a guide of primary voltage selection. Scale bar, 50 nm.

Monte Carlo simulations of BSE images provide a numerical basis for modeling sub-surface contrast from heavy-atom stained structures in a light element matrix. We consider the model structure, shown in Fig. 2a, consisting of an 800 nm × 800 nm × 800 nm block of epoxy resin containing non-overlapping 50 nm × 50 nm × 12.5 nm cuboids spaced 50 nm apart in the *x*-direction and 12.5 nm apart in the *z*-direction, in each of which has been added 3 atomic percent of lead. From the known composition and density of epoxy resin (Supplementary Table 1), the stain density in the cuboids is 3.24 lead atoms per nm^3^. This lead concentration was chosen to be commensurate with the maximum experimental level of staining used in SBEM (see Methods).

To extract information about the variation of stain as a function of depth below the block surface, we consider two BSE images acquired at primary energies, *E*_*low*_ and *E*_*high*_. The choice of primary energies for recording the BSE images depends on the density of the low atomic number matrix, which determines the inelastic mean free path. Since the BSE signal depends not only on the depth of a stained structure below the block face, but also on the numbers of heavy atoms that it contains, determination of the feature depth requires consideration of the BSE signal ratio for energies *E*_*low*_ and *E*_*high*_.

Monte Carlo simulations of BSE intensities were performed for primary beam energies ranging from 0.8 keV to 2.9 keV at intervals of 0.1 keV for the model structure (Fig. 2a). BSE images for each primary energy are displayed in Fig. 2b, and histograms of the mean intensities for each cuboid are indicated in Fig. 2c. It is evident that cuboids situated at greater depths from the surface do not contribute to the BSE signal until the beam energy reaches a certain value. For example, at beam energies of 0.8 keV and 0.9 keV, only the cuboid closest to the surface within a 12.5 nm layer (Fig. 2c, blue bar) makes a significant contribution to the backscattered signal. At a beam energy of 1.0 keV, the cuboid situated in the layer from 12.5 nm to 25 nm below the surface begins to contribute to the BSE signal (Fig. 2c, red bar), and becomes a major component at beam energies of 1.3 keV and 1.4 keV. At a beam energy of 1.4 keV, the cuboid situated in the layer from 25 nm to 37.5 nm below the surface begins to contribute to the BSE signal (Fig. 2c, green bar), and becomes a major component at beam energies of 1.7 keV and 1.8 keV. At a beam energy of 1.8 keV, the deepest cuboid in our model structure situated in the layer from 37.5 nm to 50 nm below the surface begins to contribute to the BSE signal (Fig. 2c, purple bar), and becomes a major component at a beam energy of ~2.2 keV. It is evident that the BSE signal from cuboids located further away from the surface rarely exceed the signal from the cuboid at the surface, which makes it possible to separate two layers by analyzing two images taken at appropriately chosen beam voltages. In practice, the lowest primary beam energy is limited by factors including microscope working distance, detector sensitivity and sample stability under electron irradiation.

For the Zeiss Sigma VP SEM equipped with a Gatan 3 View serial block-face system used in the present study, the lowest practicable beam energy is *E*_*low*_ = 1.0 keV, which provides a BSE signal almost entirely from the 12.5 nm layer immediately beneath the block face; and by selection of a second beam energy *E*_*high*_ = 1.4 keV, gives a BSE signal in which the top two layers (0 to 12.5 nm, and 12.5 to 25 nm beneath the block face) contribute almost equally. In our simulations, we consider the following parameters: incident number of electron per pixel *J*_0_ = 10^4^ electrons; heavy atom stain concentration, ${\gamma }_{stain}$ = 3% in the cuboids; and BSE detector efficiency, ${{\epsilon }}_{det}$ = 1.0 (see Supplementary Table 1).

**Figure 3:**
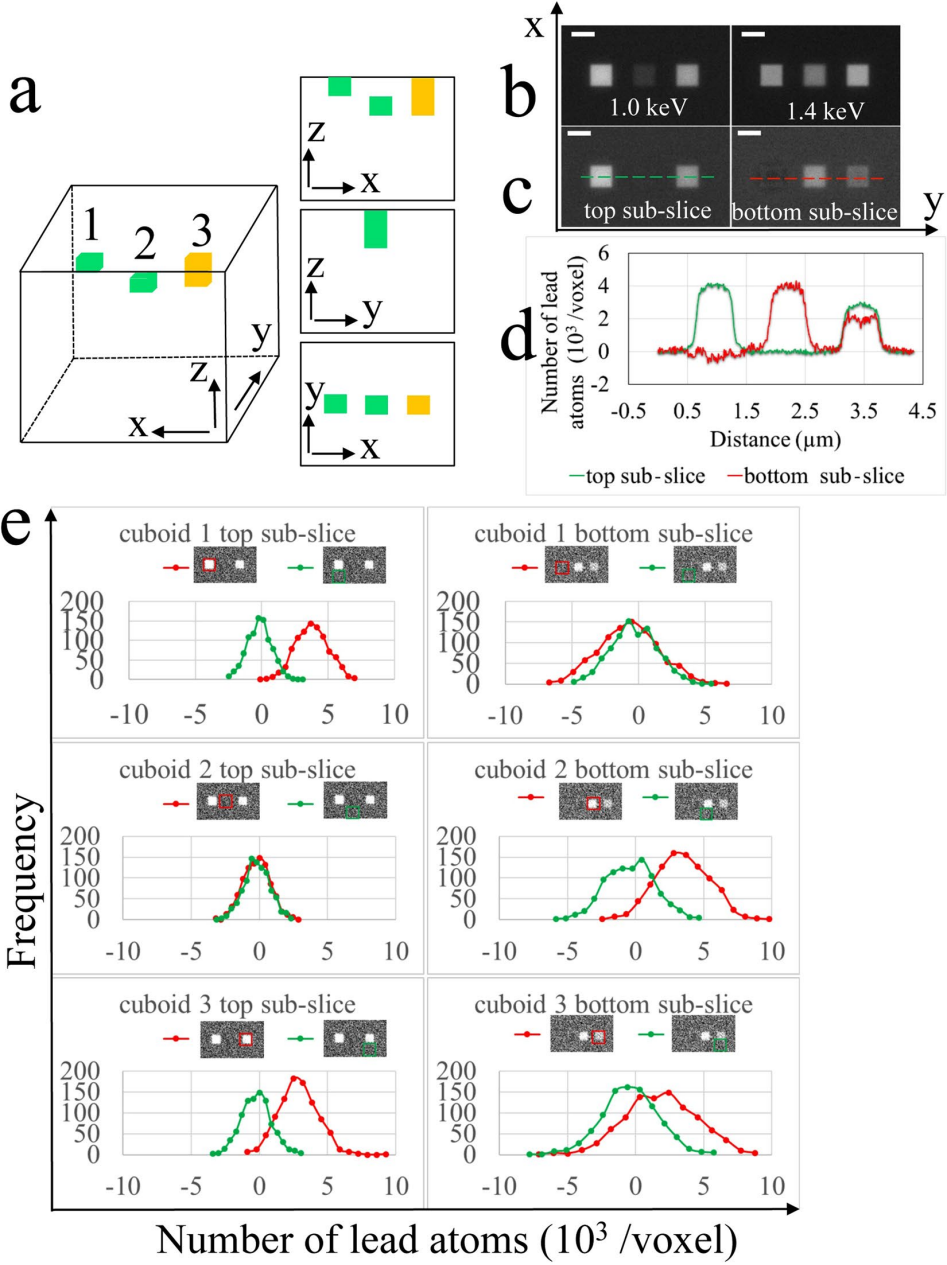
Effect of stain composition on accuracy of the methods. (**a**) Three-dimensional geometrical model for two 625 nm × 625 nm × 12.5 nm cuboids (green) stained with 3% lead and containing 2,500 voxels of dimensions 12.5 nm × 12.5 nm × 12.5 nm, and one 625 nm × 625 nm × 25 nm cuboid (yellow) stained with 1.5% lead and containing 5,000 voxels of the same dimension, located at different depths in a 10 µm × 10 µm × 10 µm epoxy block, with views in the *x*-*z*, *y*-*z* and *x*-*y* planes. Centers of cuboids are located at 6.25 nm, 18.75 nm and 12.5 nm, respectively, from the top surface of the block (left to right). Dimension in *z* is not drawn to scale. **(b)** Simulated images at primary beam energies of 1.0 keV and 1.4 keV; and **(c)** calculated sub-slice specimen structures with a nominal z-resolution of 12.5 nm. **(d)** Line profile of the features in the top and bottom sub-slices are indicated by dashed lines in **(c)**. **(e)** Histograms for cuboids 1 to 3 and their corresponding backgrounds in the top and bottom sub-slice at a fluence of 1000 e/pixel. Scale bar, 625 nm.

The intensities of the cuboids for the images at both landing energies are determined after first subtracting the background, which gives the coefficients of the 2 × 2 matrix:

7 $${\bf{A}}=(\begin{array}{cc}1.043\times {10}^{-3} & 0.130\times {10}^{-3}\\ 0.898\times {10}^{-3} & 0.648\times {10}^{-3}\end{array})$$

where the coefficients of matrix **A** have units of nm^2^. The inverse of the matrix **A** is given by:

8 $${{\bf{A}}}^{-1}=(\begin{array}{cc}1.166\times {10}^{3} & -\,0.227\times {10}^{3}\\ -\,1.620\times {10}^{3} & 1.879\times {10}^{3}\end{array})$$

where the coefficients of matrix **A**^−1^ have units of nm^−2^. To test the sensitivity of the algebraic computation of stain density based on Equation 6, a model structure was generated (Fig. 3a) with two cuboids of dimensions 625 nm × 625 nm × 12.5 nm (containing 2,500 voxels of dimension 12.5 nm × 12.5 nm × 12.5 nm) with a lead concentration of 3% (cuboid 1 and cuboid 2) and one cuboid of size 625 nm × 625 nm × 25 nm (containing 5,000 voxels of dimension 12.5 nm × 12.5 nm × 12.5 nm) with a lead concentration of 1.5% (cuboid 3). These three cuboids contain the same total amount of stain, but cuboid 3 has half the stain density of cuboid 1 and 2. Figure 3b shows the calculated backscattered images at 1.0 keV and 1.4 keV for a total fluence of 13 e/nm^2^ per 12.5 nm × 12.5 nm pixel, corresponding to 1,000 electrons per pixel for each image to match the experimental conditions.

Using Equation 6, we can determine the sub-slice stain density, as shown in Fig. 3c, and thereby obtain an improved z-resolution of 12.5 nm. Now it is evident that each green cuboid in Fig. 3a containing 3% lead appears in its proper sub-slice, and no stain appears in the other sub-slice. Furthermore, the yellow cuboid in Fig. 3a containing half stain concentration (1.5% lead) and extending over 25 nm in the *z*-direction, correctly appears in both sub-slices. This result demonstrates that the algebraic computation is sufficiently sensitive to determine the depths of small stained features contained in a specimen block.

The sub-slice reconstruction inevitably introduces some noise, which can be attributed to subtraction of cross terms in the matrix multiplication, and which is particularly evident in the lightly stained cuboid 3 in Fig. 3d. The histograms of computed numbers of lead atoms per voxel in Fig. 3e show that peaks of the voxel values from both cuboids 1 and 2 are situated at ~4,000 stain atoms, whereas the peaks for the neighboring unstained embedding material are centered near zero atoms of stain, as expected. The separation of voxel values between cuboid 3 and the neighboring unstained background is ~2,000 lead atoms in both sub-slices of the cuboid, which is consistent with the model structure. However, the full-width of the peak in the voxel histogram of ~3,000 lead atoms implies that it is more difficult to separate features at a nominal z-resolution of 12.5 nm with a low stain concentration at a fluence of ~13 e/nm^2^, which is close to the experimentally determined maximum permissible fluence to obtain regular cutting in our SBEM for specimens prepared according to the standard NCMIR protocol.^12^ Despite the limitations indicated above, our computational model for sub-slice structure determination is predicted to provide a substantial advantage in *z*-resolution.

### Imaging liver sample with a factor of two improvement in z-resolution

Next, we applied our sub-slice reconstruction scheme to visualize membranes of hepatocytes in a test sample of mouse liver that had been stained with heavy metals and embedded in epoxy resin, according to the NCMIR protocol^12^. Two BSE image sets were collected at different cutting intervals. One image set includes 79 consecutive cuts at 50 nm increments, with primary beam energies of 1.4 keV and 2.2 keV, and a pixel size of 12.5 nm with a total fluence of 20 electrons/nm^2^. The other image set consists of 73 consecutive cuts at 25 nm increments, with primary beam energies of 1.0 keV and 1.4 keV, and a pixel size of 12.5 nm with a total fluence of 13 electrons/nm^2^. Since the effect of beam damage is somewhat reduced for larger cutting increments, the allowable dose for block face imaging at 50-nm increments is slightly higher than for block face imaging at 25-nm increments. The purpose of showing two datasets at different cutting intervals is to test that our computational model has the versatility to extract sub-slice information at different depths according to the choice of primary energies. The matrix **A** for 50-nm cutting increments and 25-nm sub-slice thickness is presented in Supplementary Note 1, and the matrix **A** for 25-nm cutting increments and 12.5-nm sub-slice thickness is gven by Equation 7.

Sub-slice structure was obtained by applying the reconstruction technique to the noise-reduced datasets obtained from the liver samples. A selection of four sequential block face images at cutting increments of 25 nm is shown in Supplementary Fig. 3. The calculated sub-slices appear noisier than the raw acquired images at primary energies E_low_ and E_high_, as expected from the application of the inverse matrix in Equation 2. Nevertheless, the calculated dataset at twice the z-resolution exhibits fine features that would be missed without using sub-slice imaging technique (Supplementary Fig. 3).

**Figure 4:**
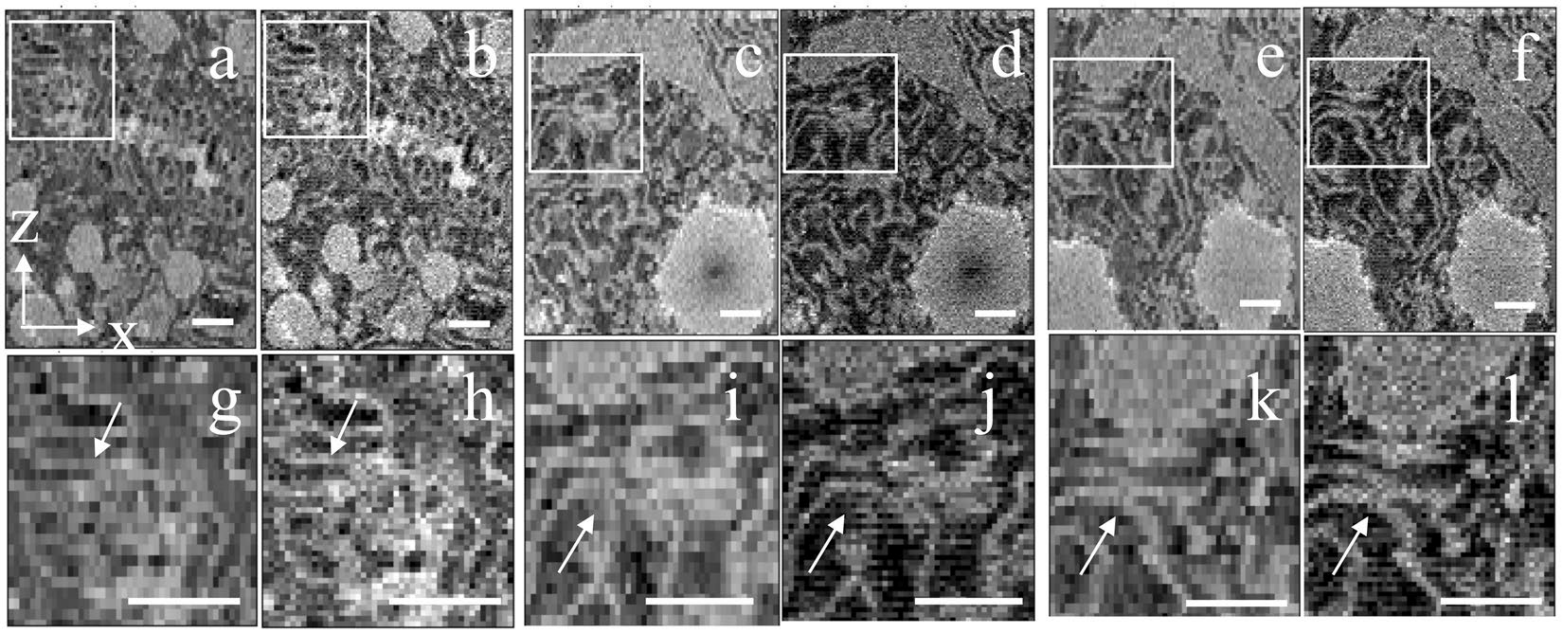
Reconstructed volume in *x*-*z* plane from liver sample cut at increments of 50 nm and imaged with primary energies of 1.4 keV and 2.4 keV. (**a**,**c**,**e**) Average of BSE image stacks acquired at beam energies of 1.4 keV and 2.4 keV, shown in three different regions of hepatocyte. (**b**,**d**,**f**) Same views as in (**a**,**c**,**e**) but calculated with sub-slice *z*-resolution. (**g**,**i**,**k**) Views at higher magnification from the rectangular regions indicated in (**a**,**c**,**e**), respectively. (**h**,**j**,**l**) Views at higher magnification from the rectangular regions indicated in (**b**,**d**,**f**), respectively. Arrows indicate membranes of endoplasmic reticulum that show higher resolution in the sub-slice reconstruction. Scale bar, 500 nm.

To evaluate the *z*-resolution improvement, *x*-*z* and *y*-*z* views were generated from block face images before and after applying the sub-slice technique to an image stack cut at 50-nm increments. Figure 4a,c,e are the *x*-*z* views of averaged images acquired at E_low_ = 1.4 keV and E_high_ = 2.2 keV, which enable us to compare 3D volumes generated without the sub-slice technique but with the same total electron fluence. Views at higher magnification in Fig. 4g,i,k show rectangular pixels, in the absence of the sub-slice computation. Figure 4b,d,f are the corresponding *x*-*z* views after sub-slice analysis with a resulting *z*-resolution of 25 nm, and views at higher magnification in Fig. 4h,j,l now display square pixels, after sub-slice analysis. A comparison of *x*-*z* views generated with and without sub-slice analysis demonstrate a substantial improvement in spatial resolution using the sub-slice computation for features that are oriented with a strong component in the *x*-direction or orthogonal to the *z*-direction. This is evident in the membranes of endoplasmic reticulum (ER) in the hepatocytes.

**Figure 5:**
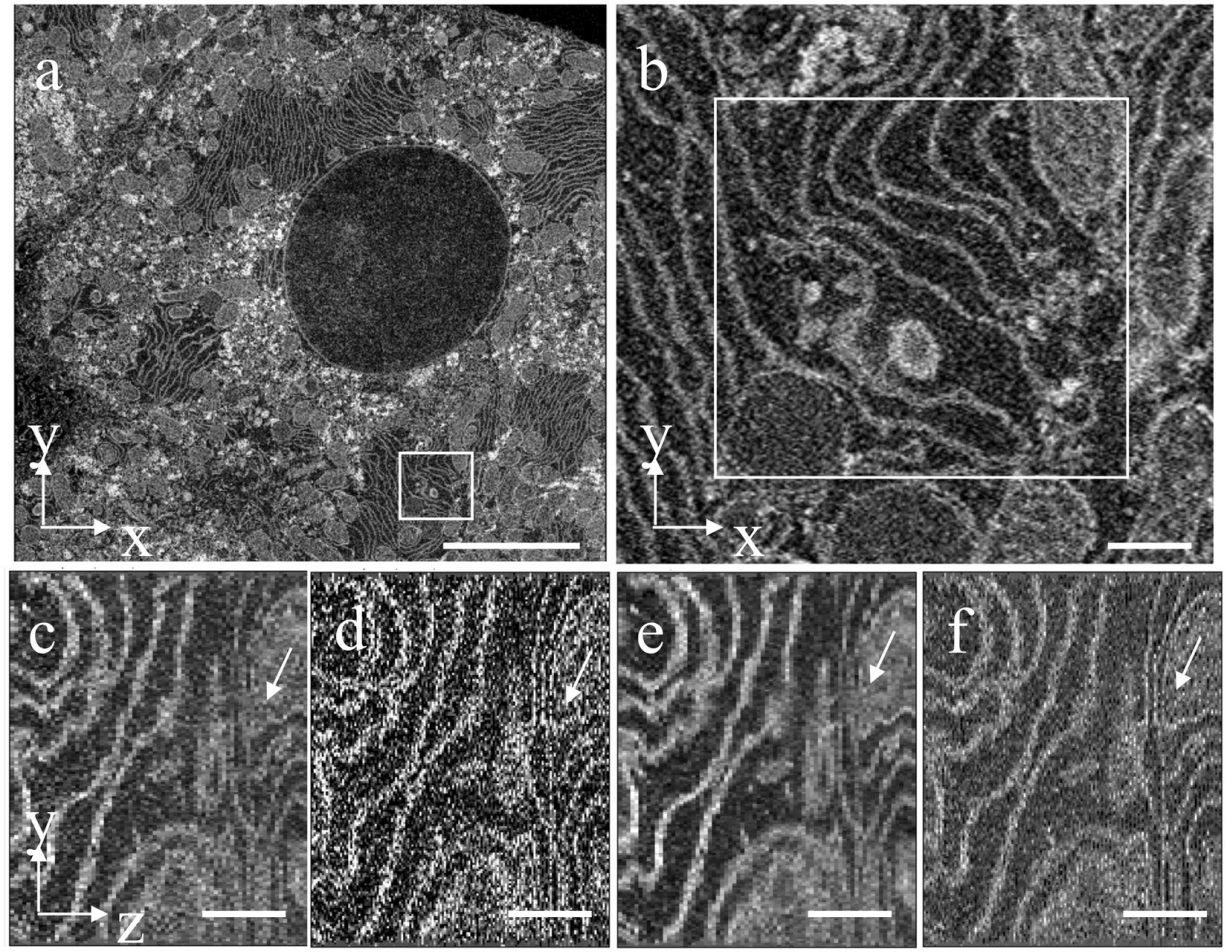
Reconstructed volume in *x*-*y* and *y*-*z* planes from liver sample cut at increments of 25 nm and imaged with primary energies of 1.0 keV and 1.4 keV. (**a**) Low-magnification BSE image of hepatocyte in the *x*-*y* plane acquired at primary energy of 1.4 keV. (**b**) Region indicated by square in (**a**) at higher magnification showing membranes of endoplasmic reticulum. (**c**) Average of 1.0 keV and 1.4 keV BSE image intensities displayed in the *y*-*z* plane. (**d**) Calculated sub-slice stack in the *y*-*z* plane showing improved spatial resolution relative to (**c**), but with additional noise. (**e**) Five-slice average in the *x*-direction of *y*-*z* view in (**c**). (**f**) Five-slice average in the *x*-direction of *y*-*z* view in (**d**). Arrows in (**c–f**) denote areas with improved resolution. Scale bar in (**a**), 5 µm; and in (**b–f**), 500 nm.

**Figure 6:**
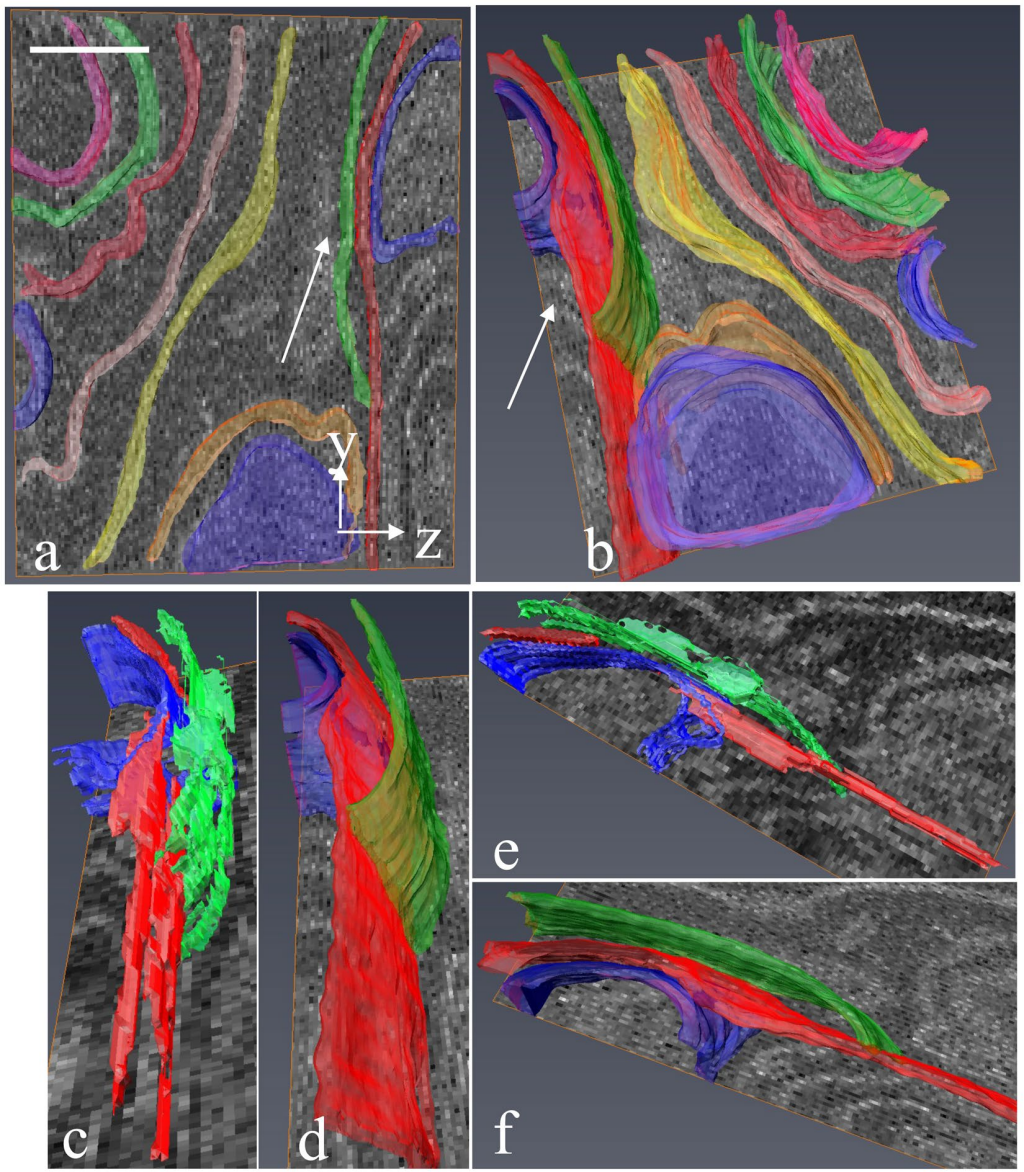
3D rendered membranes with and without sub-slice reconstruction. (**a**,**b**) Views of region of hepatocyte in the *y*-*z* orthoslices of Fig. 5(d,f) show clear separation of membranes after sub-slice reconstruction. **(c**,**e)** Views of three membranes indicated by arrow in **(a**,**b)** obtained from the averaged image stacks acquired at beam energies of 1.0 keV and 1.4 keV, i.e., without sub-slice reconstruction, showing that it is not possible to segment the green and red membranes accurately due to the limited *z*-resolution of 25 nm in the individual datasets acquired at each beam energy. **(d**,**f)** Same views as in **(c**,**e)** but after sub-slice reconstruction, again showing a clear separation of the green and red membranes. Scale bar, 500 nm.

Next, the sub-slice technique was applied to a sample block of mouse liver using cutting increments of 25 nm and BSE images acquired at primary beam energies of *E*_*low*_ = 1.0 keV and *E*_*high*_ = 1.4 keV. Figure 5a is an *x*-*y* view of the block face showing a region of endoplasmic reticulum in a hepatocyte. An orthogonal view in Fig. 5b shows a *y*-*z* view of the same region obtained without sub-slice analysis and with the averaged intensities of the image stacks acquired at *E*_*low*_ = 1.0 keV and *E*_*high*_ = 1.4 keV, i.e., with a dose corresponding to that used for sub-slice analysis. Despite some additional noise, it is evident that regions of ER indicated by the white arrows in the sub-slice image stack (Fig. 5c) appear sharp with one-pixel wide membranes that are well separated compared with the same structures in Fig. 5b. The signal-to-noise ratio in the *y*-*z* plane can be improved by averaging several *y*-*z* images at different *x*-values. Five-slice averages along the *x*-axis of the *y*-*z* views in Fig. 5b,c are shown in Fig. 5d,e, respectively; the improvement in z-resolution obtained by sub-slice analysis for the ER membranes oriented close to the *x*-*y* plane is now seen more clearly. A 3D surface rendered model of the membranes contained in the *y*-*z* views in Fig. 5b,c are presented in Fig. 6a,b, and the three closely spaced ER membranes indicated by the arrow in Fig. 5c–f are shown in Fig. 6c,e without the sub-slice analysis and in Fig. 6d,f after sub-slice analysis. In the 3D visualization of the image stacks processed with the sub-slice analysis (Fig. 6a,b,d,f), the membranes shown in red and green are clearly separated, whereas it is difficult to trace the same membranes in the image stack without sub-slice analysis (Fig. 6c,e). A visualization of the membranes is also presented in Supplementary Movie 1.

## Discussion

Previously a technique has been developed to determine subsurface structure in SBEM by acquiring images at different primary beam energies and then by processing the image stacks using a proprietary blind deconvolution multi-energy deconvolution algorthim (Teneo VolumeScope)^22–24^. We have developed a complementary approach based on a quantitative physical Monte Carlo model of electron scattering^27–30^, which provides an objective measure of the capabilities and limitations of the technique as a function of operating parameters of the SEM, BSE detector, ultramicrotome, and the specimen composition. This framework provides a way of assessing the capabilities and limitations of SBEM for addressing specific biological problems.

We do not attempt here to evaluate rigorously the improvement in *z*-resolution that can be realized with using the multiple landing energy approach, and a proper measure of *z*-resolution in either SBEM or FIB-SEM has yet to be established. We do know, however, that using a single landing energy, the *z*-resolution in our SBEM is limited to 25–30 nm by the minimum cutting thickness, and we believe that we have demonstrated a factor of two improvement using dual beam energy acquisition, but we have not determined whether it is possible to improve the spatial resolution beyond this factor of two by acquiring further images at other beam energies. The ultimate z-resolution in FIB-SEM depends on the interaction volume, as discussed by Xu *et al*.^31^, which shows that a z-resolution of ~5 nm in the FIB-SEM is only achievable with a low landing energy of around 0.8 keV. Our Monte Carlo simulations suggest that published results from FIB-SEM datasets acquired using landing energies of 1 keV have *z*-resolutions ≳1 nm, and datasets acquired using landing energies of 1.5 keV have *z*-resolutions ≳25 nm. In our SBEM experiments, the highest resolution that we have demonstrated is ~12 nm. Moreover, there appears to be no advantage in imaging the layer immediately below the block face at 5-nm *z*-resolution if the minimum cutting thickness is 25 nm. However, in the FIB-SEM, it would presumably be possible to image the block face with dual landing energies of ~0.8 keV and ~1.0 keV by removing successive 10-nm layers with the FIB, while maintaining ~5-nm *z*-resolution throughout the 3D volume.

Another limitation in our SBEM is difficulty in operating with landing energies below 1.0 keV due to the minimum available working distance, which is restricted by the space needed to accommodate the in-chamber ultramicrotome. This determines the lowest usable primary beam energy for acquiring BSE images that are well focused; in our system, the minimum usable energy E_low_ is 1.0 keV, even though our Monte Carlo simulations predict that a primary beam energy of 0.9 keV would provide a higher *z*-resolution (as demonstrated in Fig. 2b,c). Recently, there has been interest in using stage deceleration for SBEM with the purpose of keeping the beam voltage and the electron optical conditions of the probe-forming lenses constant, while applying a stage deceleration to achieve a focused probe at lower electron landing energies^32,33^.

We have shown that the matrix approach can give a factor of ~2 improvement in *z*-resolution in 3D ultrastructural data acquired in the SBEM by imaging the block face at dual primary beam energies. In principle, it is possible to achieve higher *z*-resolution by acquiring block face images at more than two primary beam energies. However, in practice, the *z*-resolution is limited by several factors, including: damage of the sample block under electron irradiation, detector quantum efficiency, detector collection solid-angle, available range of primary beam energies, and maximum uptake of heavy-atom stains by the biological structures of interest.

The most important limitation is the maximum number of electrons per unit area incident on the block face —a fluence of approximately 15–20 electrons/nm^2^— above which the sample shrinks, causing non-uniform cutting. For a pixel size (12.5 nm × 12.5 nm), which we have been considered here, the total number of incident electrons per pixel in each of the dual images is approximately 1,000 electrons, and the Monte Carlo simulations predict the amount of shot noise in these images. The sub-slice computation amplifies the noise level due to subtraction of cross-terms in the matrix multiplication. Furthermore, the annular BSE detector adds noise to the images acquired at the different primary beam energies due to the limited solid angle of the detector and electronic noise in the detector.

A problem often encountered in SBEM operated in high vacuum mode is caused by subtle electrical charging effects, e.g., producing image jitter, when the primary electrons are incident on areas of the block face that are less electrically conductive^9,34^. Although the image jitter might not be obvious to the operator, it poses considerable challenges for sample alignment, which must often be performed with single pixel precision. For sub-slice imaging in the SBEM, it is not feasible to operate the SEM in the variable pressure mode, because the landing energy is not well defined due to scattering by gas molecules introduced into the SEM column. However, it has been recently reported that specimen charging can be completely avoided with charge compensation by focal gas injection of gas through a needle into the immediate vicinity of the specimen block^35^. Another possible solution for solving the electrical charging problem is the use of conductive resins^34^.

Existing sample preparation methods for SBEM, particularly the NCMIR protocol^12^, work well when imaging with one beam energy, but it would be advantageous to increase the BSE signal further for sub-slice imaging with dual beam energies. One way to achieve this would be to incorporate even higher concentrations of heavy atom stains into the specimen, and different protocols can be readily assessed using electron scattering measurements^36^. Alternatively, improved results could be obtained if embedding resins were available that are more resistant to shrinkage under electron irradiation^37^.

It is anticipated that advances, such as the putative ones mentioned above, will improve the performance of the still relatively young technique of SBEM to provide cellular ultrastructure at an isotropic sub-slice resolution of ~12 nm from image stacks that exhibit lower noise levels than are feasible with our present instrument. Such a capability will be valuable, for example, in neuroscience applications, where it could enable the visualization of individual presynaptic vesicles in nerve terminals, as well as improved tracing of neuronal circuitry with higher precision than is now achievable.

## Methods

### Animal preparation

Mouse liver tissue was a gift from Drs. Rodney L. Levine and Lo Lai, National Heart, Lung, and Blood Institute (NHLBI), NIH. Procedures for obtaining the tissue were approved by the NHLBI Animal Care and Use Committee (Protocol #H0120) and performed in accordance with the guidelines described in the Animal Care and Welfare Act (7 USC 2144).

### Specimen preparation

Sample blocks of mouse liver were prepared for SBEM using the NCMIR protocol^12^. Pieces of liver were dissected from mice and were fixed in a mixture of 2.5% glutaraldehyde and 2% formaldehyde in sodium cacodylate buffer with 2 mM calcium chloride at room temperature for 5 minutes, and for an additional 2–3 hours on ice in the same solution. Samples were washed 3 times with cold cacodylate buffer containing 2 mM calcium chloride and post-fixed for 1 hour in a reduced osmium solution containing 2% osmium tetroxide, 1.5% potassium ferrocyanide 2 mM CaCl_2_ in 0.15 mM sodium cacodylate buffer (pH 7.4), followed by incubation with a 1% thiocarbohydrazide (TCH) solution in ddH_2_O for 20 minutes at room temperature (RT). Subsequently, samples were fixed with 2% OsO_4_ in ddH_2_O for 30 minutes at room temperature, followed by 1% aqueous uranyl acetate at 4 °C for 12 hours. The samples were then subjected to *en bloc* Walton’s lead aspartate staining^38^, and placed in a 60 °C oven for 30 minutes. After dehydration in graded concentrations of ice-cold anhydrous ethanol for 5 minutes each, the samples were placed in anhydrous ice-cold acetone at room temperature for 10 minutes. The pieces of tissue were infiltrated with 30% Hard-Plus for 2 hours, 50% Hard-Plus for another 2 hours, 75% Hard-Plus overnight, and 100% Hard-Plus for one day before being polymerized in a 60 °C oven for 48 hours. The polymerized blocks were mounted onto special aluminum pins for SBEM imaging. Sample blocks that were imaged with 50-nm cutting increments were prepared with the Hard-Plus embedding resin; sample blocks that were imaged with 25-nm cutting increments were prepared (by Gatan Inc.) using the same procedure as above but with Durcupan embedding resin.

### Estimation of stain content of hepatocytes in specimen of liver tissue

We measured the stain concentration in the block by removing small ~1 mm^3^ pieces of heavily stained liver tissue from the resin-embedded blocks and determined their density, by floating them in a tube of concentrated lead nitrate solution, and then adding water until the pieces precipitated rapidly to the bottom of the tube. The density of the stained block was found to be 1.51 ± 0.01 g/cm^3^, compared with a density of 1.22 ± 0.01 g/cm^3^ for the unstained epoxy resin block. The stained blocks therefore contain 0.24 ± 0.02 grams of stain per gram of resin. Considering that the stain is composed of Os, Pb, and U, and that the composition of epoxy resin is 36 at. % C, 52 at. % H, and 12 at. % O, it was determined that the average total concentration of heavy atoms in the highly stained liver specimen is only ~1.0 atomic percent. Furthermore, by analyzing the contrast in the backscattered electron images, it was found that even the most highly stained structures in the specimen produce a BSE signal that is only a factor of three greater than the average BSE signal, so that we deduce that the most highly stained structures only contain 3 at. % heavy atoms. Experience with many different types of specimens prepared for SBEM show that such a specimen composition is typical. Data in Supplementary Fig. 5 demonstrate that the BSE signal is nearly proportional to heavy-atom stain content for concentrations below 10 atomic percent.

### Monte Carlo simulation

Image simulation was performed using the CASINO program^29,39^, which is a 3D Monte Carlo software that includes a user-friendly interface and efficient code using advanced physical modeling of electron scattering^27–30^, making it suitable for applications to electron microscopy. Total and partial cross-sections in the Monte Carlo simulation were based on the Elastic Scattering of Electrons and Positrons by Atoms (ELSEPA) formulation and database, which incorporates a Dirac partial-wave extension to a modified Mott differential cross section for elastic scattering^40^. The Monte Carlo simulation uses a stopping power equation, as previously described^41^. Before running the simulations, a 3D model of the specimen is created (Fig. 2a). In the first simulation, the model consisted of an epoxy block containing 8 lead stained (3%) cuboids of dimensions 50 nm by 50 nm by 12.5 nm and density 1.52 g/cm^3^ located at different depths inside the block. The block volume was defined to have dimensions of 800 nm by 800 nm by 800 nm, with the block size deliberately selected to be much larger than the area of interest to avoid edge effects. The z-coordinate for the top surface of the block was defined to be at zero. The z-coordinate for the mass center of the cuboids ranged from 6.25 nm to 93.75 nm with adjacent cuboids being spaced 12.5 nm apart. The epoxy block consisted of epoxy resin (Epon 812) of density 1.22 g/cm^3^ and containing hydrogen, carbon and oxygen with atomic fractions n_H_ = 0.53, n_C_ = 0.35, and n_O_ = 0.12. The cuboid size and adjacent distance were chosen to provide a realistic standard for biological specimens with the cuboids containing lead stain at a concentration of 3 atomic percent. The rationale for the choice of lead as the stain was based on the experimentally determined composition of stained specimens prepared for SBEM (Supplementary Fig. 4), and the stain concentration of 3 atomic percent is lower than that used in the Monte Carlo simulations of Hennig and Denk^25^, who demonstrated that image contrast was in the linear range for osmium at an atomic concentration of 12 atomic percent or below. Microscope parameters used for Monte Carlo simulation are listed in Supplementary Table 1. In the image simulations, we only consider the backscattered electron signal.

### Serial block face imaging

Liver samples mounted on aluminum pins were inserted into the specimen stage of a Sigma-VP (variable pressure) SEM (Zeiss Inc.) equipped with a 3View serial block face imaging system (Gatan Inc). The same area of a sample was imaged at the primary energies, *E*_*low*_ and *E*_*high*_. The SEM was operated in high vacuum mode using a condenser aperture of diameter 30 µm. For a cutting increment of 25 nm and with a probe current of 75 pA, pixel dwell times of 3.5 and 1.5 µs per pixel were selected for imaging at primary beam energies of 1.0 keV and 1.4 keV, respectively. For a cutting increment of 50 nm and with a probe current of 48 pA, pixel dwell times of 7.0 and 3.5 µs per pixel were selected for imaging at primary beam energies of 1.4 keV and 2.2 keV, respectively. For each cut of the block face, the BSE image with lower landing energy was acquired before the image with higher landing energy. No differences in image quality or computed stain distributions were observed when the order of image acquisition was reversed. Image dwell times and pixel sizes were selected to provide appropriate values of the electron fluence that gave the highest image quality, while maintaining uniform cutting of the sample. The electron fluence on the sample block was 15 e/nm^2^ for 25-nm cutting increments, and 20 e/nm^2^ for 50-nm cutting increments.

### Acquisition script

A custom script written for Digital Micrograph (Gatan Inc.) was used for dual energy imaging. This script is included in Supplementary Note 2.

### Pre-processing of image stacks

First, the local background of the original BSE images was subtracted, and a coarse alignment was applied to the images in the stack by using the IMOD software (University of Colorado)^42^. Then the image alignment was refined though the application of consecutive fine alignments using the IMOD software to select successively smaller sub-regions of interest. The process was repeated six times until the desired alignment was achieved with single pixel precision. Images acquired at a cutting increment of 25 nm were assembled into a stack with alternate low and high primary energies, as illustrated in Supplementary Fig. 1. After alignment, two sub-stacks at beam energies *E*_*low*_ and *E*_*high*_ were extracted from the fine-aligned stack, and the pixel intensities of the stack acquired at beam energy *E*_*low*_ were scaled to the pixel intensities of the stack acquired at beam energy *E*_*high*_ by using the numerical values in the simulation data. To perform the scaling, we selected large structures that were assumed to extend homogeneously throughout the surface layer, as indicted by the red triangle in Supplementary Fig. 2, and we scaled the mean intensity of the pixels in homogeneous regions of the images acquired at beam energy *E*_*low*_ by (A_11_ + A_12_)/(A_21_ + A_22_) = 0.78 of the mean intensity of the pixels in the images acquired at beam energy *E*_*high*_. This scaling factor ensured that application of the inverse matrix **A**^−1^ to the experimental data gave results that were consistent with the Monte Carlo simulation. To reduce the noise in the background-subtracted image series from blocks cut at increments of 25 nm, pixel values smaller than one standard deviation of the background as well as all negative intensities were set to zero (see Supplemental Fig. 2). We further decreased the noise in the lower sub-slice structure obtained from Equation 2, by averaging the lower sub-slice structure with the mean structure from the two neighboring upper slices.

### Image processing

Sub-slice analyses, based on Equation 2, were performed using ImageJ Macro language (NIH). Post-image denoising was performed using a scripting program in Fortran.

### Data visualization

3D rendering model (Fig. 6) and movie (Supplementary Movie) were created using the Amira program (Thermo Fisher Scientific Inc.).

### Energy dispersive x-ray spectroscopy

To perform Monte Carlo simulations for an embedded block of stained tissue used in SBEM, we first determined the dominant stain component for the NCMIR preparation procedure. This was done by recording energy-dispersive x-ray spectra from thin sections of the same tissue block imaged by our SBEM. Spectra were recorded at a primary beam energy 120 keV using a Tecnai T12 TEM (Thermo Fisher Scientific Inc.), equipped with an X-Max 80 mm^2^ SDD detector and INCA microanalysis software (Oxford Instruments) (Supplementary Fig. 4).

### Spectrum processing

To quantify the spectrum, the DTSA-II program (National Institute of Standards and Technology) was used to generate reference spectra for copper, uranium, lead and osmium. We then used the multiple least linear squares subroutine in the DTSA-II program^43^ to fit the reference spectra to experimental spectra acquired from thin sections of the tissue block and to determine the composition in atomic fractions. The fitting procedure gave an R^2^ value of 0.99568. The result of spectrum analysis is presented in Supplementary Fig. 4.

## Electronic supplementary material

Supplementary Information

Supplementary Movie
