# Supplementary material for: Biological serial block face scanning electron microscopy at improved z-resolution based on Monte Carlo model
Source: Sci Rep. 2018 Aug 28;8:12985. doi: 10.1038/s41598-018-31231-w (PMC6113311; doi:10.1038/s41598-018-31231-w)
Supplement: Supplementary file 1 — Supplementary Information [file 41598_2018_31231_MOESM1_ESM.pdf]

04-25-18

Supplementary Information

Biological serial block face scanning electron microscopy at improved z-resolution based on Monte Carlo model

Q. He[1], M. Hsueh[2], G. Zhang[1], D.C. Joy[3, 4], R.D. Leapman[1*]

[1]National Institute of Biomedical Imaging and Bioengineering, NIH, Bethesda, MD, 20892, USA.

[2]Gatan, Inc., 5794 W. Las Positas Blvd, Pleasanton, CA, 94588, USA

[3]Department of Material Science and Engineering, University of Tennessee, Knoxville, TN, 37996, USA

[4]Center for Nanophase Material Sciences, Oak Ridge National Laboratory, Oak Ridge, TN, 37831, USA

[*]Corresponding author:
Richard D. Leapman
National Institute of Biomedical Imaging and Bioengineering,
Building 13, Room 3N17,
13 South Drive,
Bethesda, MD 20892, USA
Tel: 301-496-2599
Email: leapmanr@mail.nih.gov

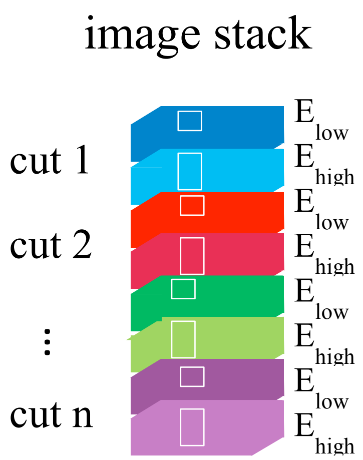

Supplementary Figure 1 | Representation of image stack used for sub-slice reconstruction. White rectangles indicate a continuous column structure that penetrates the sample. The slight shifts of columns in different cuts are due to residual charge-induced electrostatic beam deflection. Sub-slice reconstruction requires that image alignment be accurate to within a single pixel.

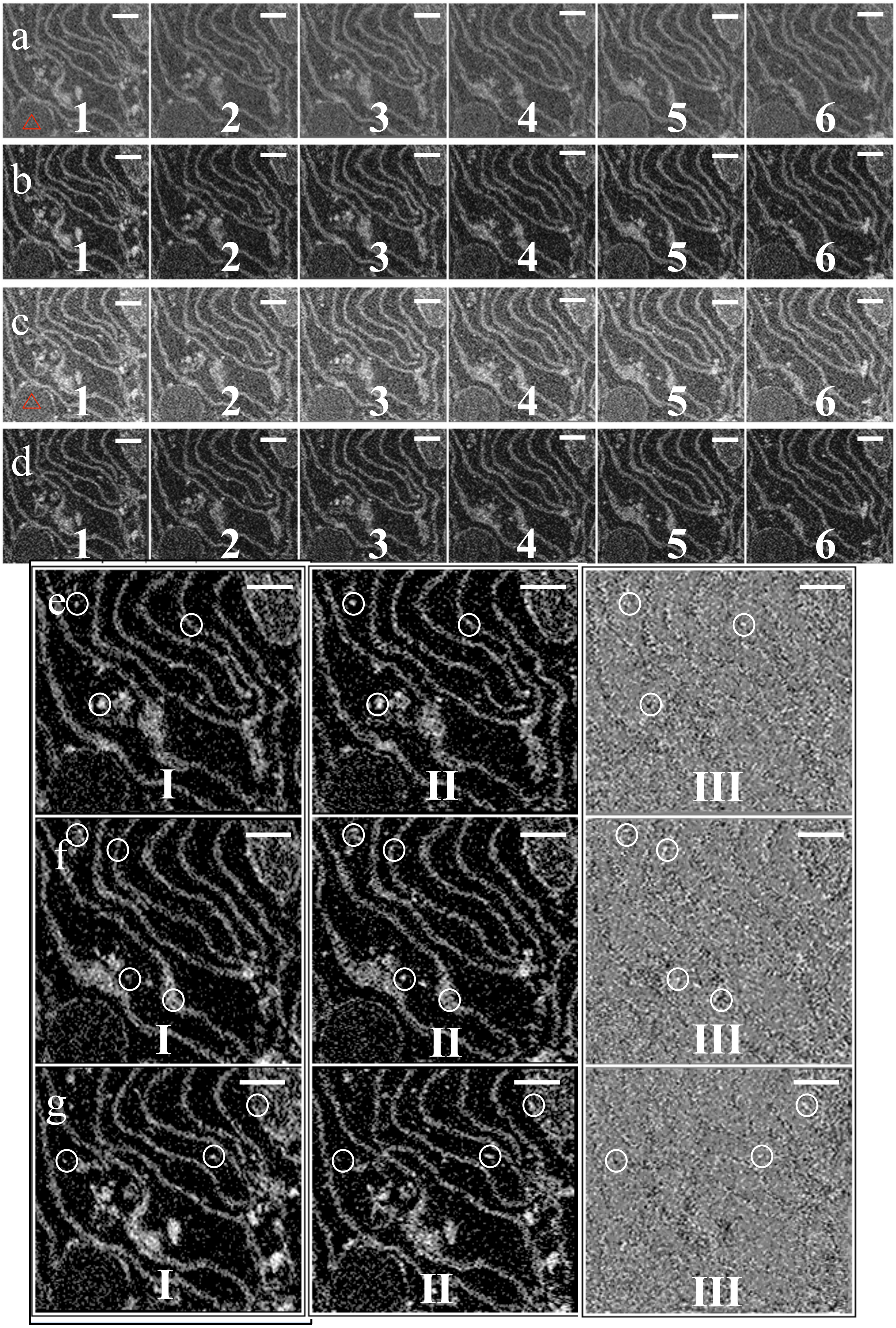
a
1
2
3
4
5
6
b
c
d
e
f
g
I
II
III

Supplementary Figure 2 | Backscattered images acquired from six successive 25-nm cuts of a liver sample that had been stained with heavy metals and embedded in epoxy resin. The block-face images are shown after fine alignment to correct for drift. (a), (c) BSE images acquired at beam energies of E_{low} = 1.0 keV and E_{high} = 1.4 keV; (b), (d) pre-processed BSE images in (a) and (c) after setting negative pixels intensities to zero. (e) (f) and (g) pre-processed BSE images and difference images of cut 1, 3 and 5 from (b) and (d) after normalizing the background. To display differences between BSE images acquired at dual landing energies, pairs of images in Figs. b1 / d1, b3 / d3, and b5 / d5 are shown at higher magnification in Figs. eI / eII, fI / fII, and gI / gII, respectively, and their differences are displayed in Figs. eIII, fIII, and gIII. White circles indicate typical differences between features, which appear bright or dark depending on whether they are located in the top or bottom subslice. Red triangles indicate homogenous region used for scaling image intensities in the E_{low} and E_{high} image stacks. Scale bar, 500 nm.

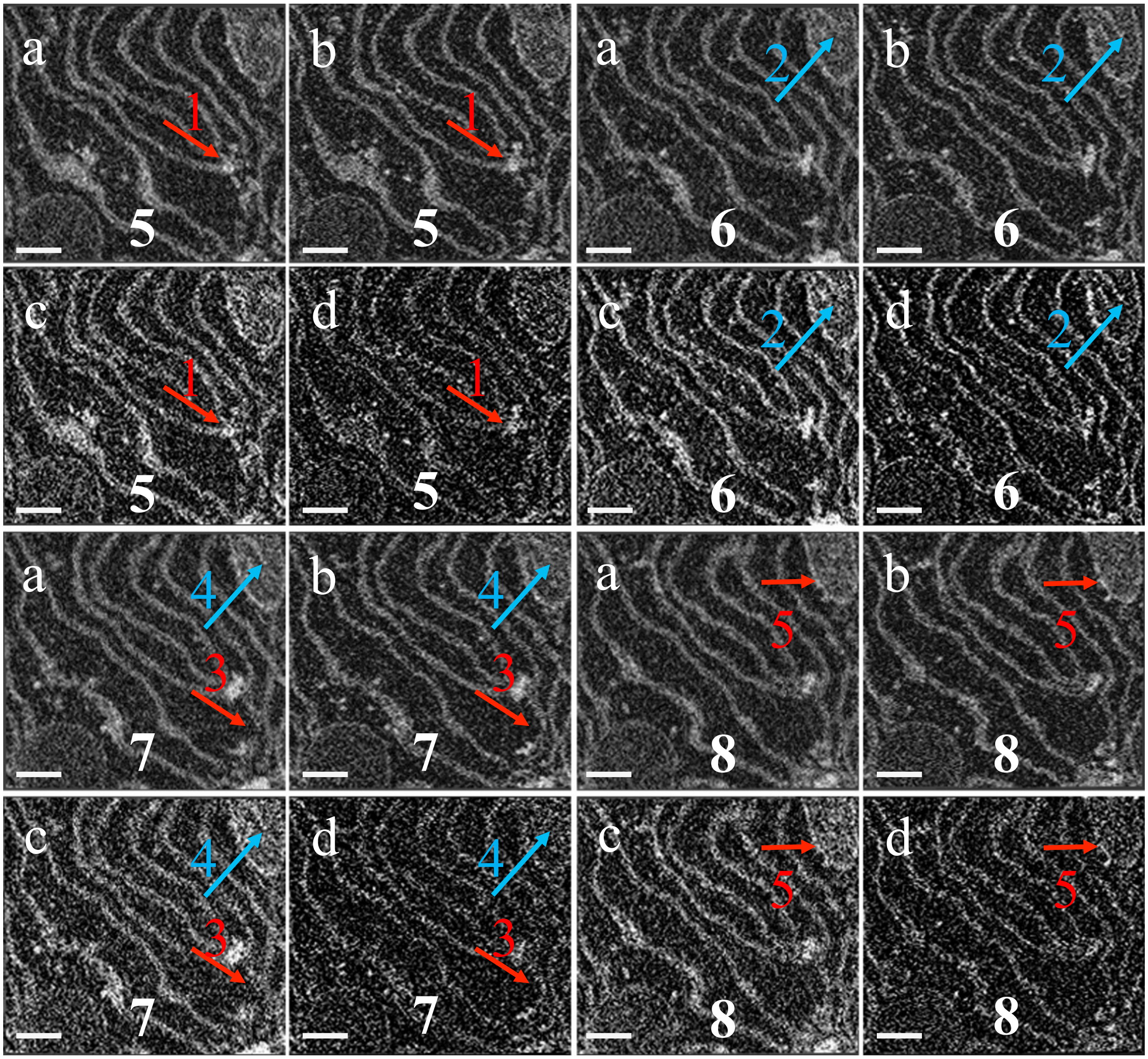

Supplementary Figure 3 | Four pairs of backscattered images at (a) E_{low} = 1.0 keV and (b) E_{high} = 1.4 keV from successive 25-nm cuts of a heavy metal-stained liver block, together with calculated structure of (c) top and (d) bottom sub-slices, each of thickness 12.5 nm. The numbers indicated in white represent the cutting order and correspond to the numbers in Supplementary Fig. 2. The numbered arrows (red and blue) denote selected fine-scale features that change from sub-slice to sub-slice. Scale bar, 500 nm.

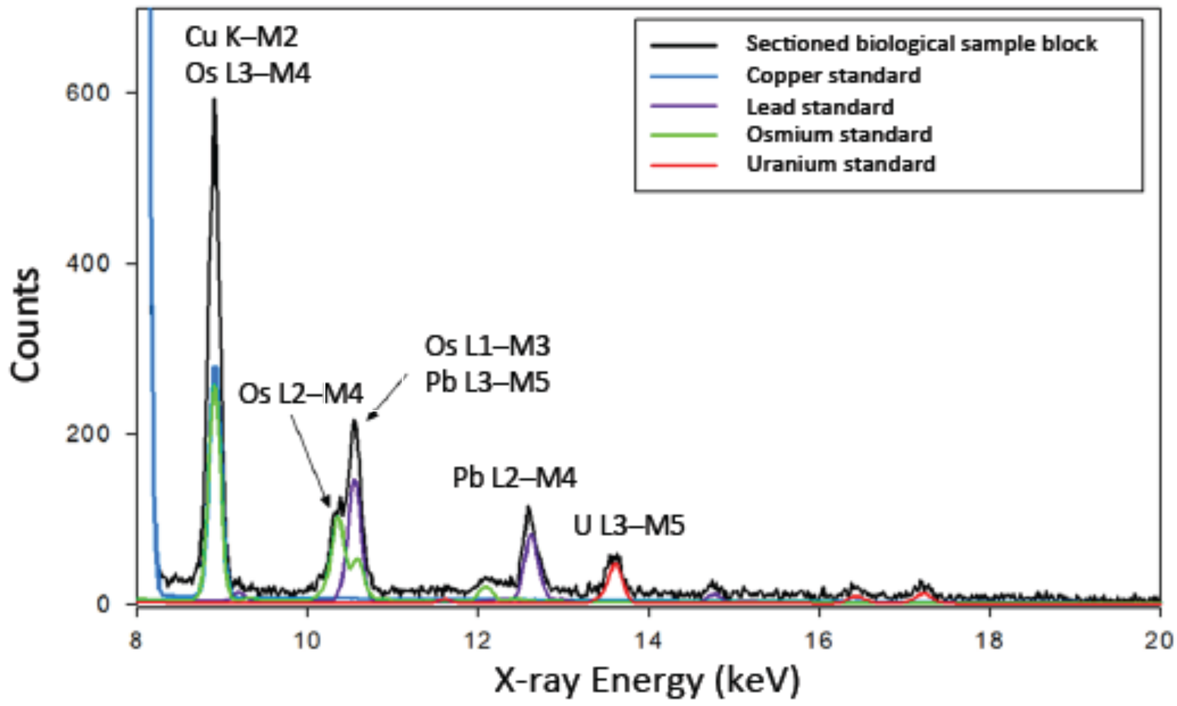

Supplementary Figure 4 | Energy-dispersive x-ray spectrum from thin section of tissue block used for SBF-SEM imaging, recorded at 120 keV beam energy using an FEI Tecnai TEM equipped with an Oxford Instruments X-Max SDD and Inca microanalysis system. X-ray spectra generated by the DTSA-II software were used as reference spectra, which were fitted to the tissue spectrum to quantitate the relative concentrations of the heavy atoms: Os, Pb, and U.

The x-ray spectrum shows that the sample contains uranium, lead and osmium, whereas the high copper peaks are due to x-ray generation in the copper grid used to mount the thin sections, and these are ignored in the analysis. There is strong overlap between the osmium L lines with copper K lines at ~ 8.9 keV, and between the lead L lines and the osmium L lines, which complicates quantification of these elements. We therefore used DTSA-II[1] to fit the spectrum, as described in Methods. The relative ratio of heavy atoms was: lead 0.0561±0.0009, osmium 0.0507±0.0003, and uranium 0.0193±0.0007. Thus, lead is the dominant stain in this specimen.

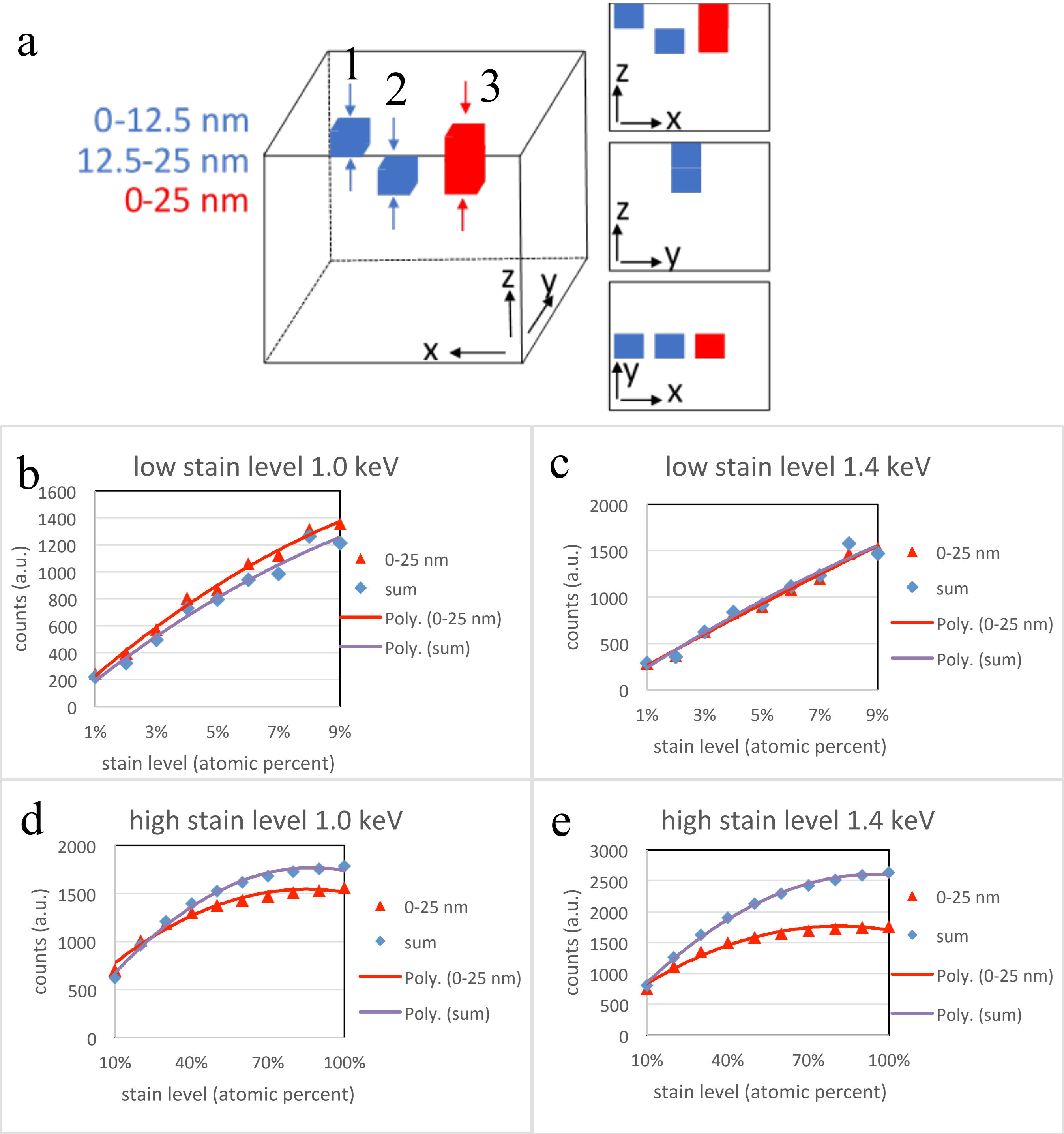

Supplementary Figure 5 |Effect of stain composition on the linearity of the backscattered signal. **(a)** Three-dimensional geometrical model for 50 nm x 50 nm x 12.5 nm lead stained cuboids located at different depths in a 800 nm by 800 nm by 800 nm epoxy block and its view of x-z plane, y-z plane and x-y plane. Centers of cuboids are located 6.25 nm, 18.75 nm, 12.5 nm, respectively, from the top surface of the block from left to right. Dimension in z is not drawn to scale. **(b)** Intensity vs. stain composition profiles for cuboids shown in (a), simulation data

were calculated at 1.0 keV with a stain composition from 1%-9%. **(c)** Intensity vs. stain composition profiles for cuboids shown in (a); simulation data were calculated at 1.4 keV with a stain composition from 1%-9%. **(d)** Intensity vs. stain composition profiles for cuboids shown in (a); simulation data were calculated at 1.0 keV with a stain composition from 10%-100%. **(e)** Intensity vs. stain composition profiles for cuboids shown in (a); simulation data were calculated at 1.4 keV with a stain composition from 10%-100%. Curves were fitted though the data using a second order polynomial regression. The red curve is based on the intensity of cuboid 3 in (a), and the blue curve is based on the sum of the backscattered signals from cuboid 1 and cuboid 2. The curves in (a) and (c) show that the backscattered signal depends linearly on stain content for concentrations below 5 atomic percent, whereas the curves in (c) and (d) reveal a strong non-linear dependence on stain content for concentrations above 10 atomic percent.

Parameter	
**Number of incident electrons / pixel**	$10,000$
**Electron beam diameter**	12.5 nm
**Pixel size**	12.5 nm
**Simulation noise**	Shot noise
**Backscattered detector efficiency (ϵ_{det})**	1.0
**Low primary energy for stained cuboids in epoxy (25 nm/cut)**	1.0 keV
**High primary energy for stained cuboids in epoxy (25 nm/cut)**	1.4 keV
**Low primary energy for stained cuboids in epoxy (50 nm/cut)**	1.4 keV
**High primary energy for stained cuboids in epoxy (50 nm/cut)**	2.2 keV
**Density of epoxy embedding resin**	1.22 g/cm^3
**Composition of epoxy resin**	$C_{36}H_{52}O_{12}$
**Heavy atom stain concentration (Pb)**	3 atomic %
**Pb stain density in cuboids**	3.24 atoms/nm^3

Supplementary Table 1. Parameters used in Monte Carlo simulation.

Supplementary Notes

Supplementary Note 1: To test the sensitivity of the sub-slice reconstruction technique, a model structure was generated containing two cuboids of size 50 nm × 50 nm × 25 nm with a lead concentration of 3% in a pure epoxy resin matrix, with one cuboid extending from the block surface to a depth of 25 nm, and the other cuboid from 25 nm below the block surface to 50 nm below the surface. Since this simulated dataset corresponded to a cutting increment of 50 nm, an electron fluence of 20 e/nm^2 was considered (i.e., somewhat higher than the maximum electron fluence of 15 e/nm^2 for cutting at increments of 25 nm). Simulated image intensities generated by the cuboids for beam energies of 1.4 keV and 2.2 keV were first processed to subtract the background, which gave the coefficients of the 2×2 matrix:

$$\mathbf{A} = \begin{pmatrix} 0.757\times10^{-3} & 0.014\times10^{-3} \\ 0.598\times10^{-3} & 0.400\times10^{-3} \end{pmatrix}$$

from which the inverse matrix can be calculated:

$$\mathbf{A}^{-1} = \begin{pmatrix} 1.359\times10^{3} & -0.048\times10^{3} \\ -2.031\times10^{3} & 2.571\times10^{3} \end{pmatrix}$$

Supplementary Note 2: DM script used to take the dual energy dataset.

```
// $BACKGROUND$
//define total cuts
number ncut = 80
number SCOPE_DELAY = 8
//number delta_x = 42.033
//number delta_y = 14.856
//number delta_beamshiftx= 13.802
//number delta_beamshifty = -20.658
number NewObstigx = 10.821
number NewObstigy = -1.683
number NewFocus =6008
number NewMag =8950
number Mag =8750
number NewStageX =-394.79
number NewStageY =-205.6
//number delta_focus = -882
//number delta_mag = 1003
//cutting and imaging loop start
for (number n = 0; n < ncut; n++)
{
EMWaitUntilReady( )
EMUpdateCalibrationState( )
Result( "\n\n\n" )
Result( " **** Basic Microscope Parameters **** \n" )
if ( !EMIsReady( ) )
{
Result( "--waiting for microscope to be ready-- \n" )
EMWaitUntilReady( )
}
Result( "--microscope is ready-- \n\n" )
EMUpdateCalibrationState( )
Result( "Microscope: " + EMGetMicroscopeName( ) + "\n" )
//read microscope high tension
if ( EMCanGetHighTension( ) )
{
Result( "High Tension: " + EMGetHighTension( ) + " Volt \n" )
}
else
{
Result( "High Tension: CAN NOT BE READ \n" )
}
```

```
Result( "Operation mode: " + EMGetOperationMode( ) + "\n" )
//read microscope magnification
if ( EMCanGetMagnification( ) )
{
if ( EMGetOperationMode( ) != "DIFFRACTION \n" )
{
Result( " -Magnification: " + EMGetMagnification( ) + "\n")
}
}
else
{
Result( " -Magnification: CAN NOT BE READ \n" )
}
// read focus and stigmation
number focus // focus
number ObStigX, ObStigY // objective stigmator
//number Brightness, Contrast
//void PrintAllValues( number calibrated )
Result( "\n\n" )
Result( "--- RAW VALUES --- \n" )
focus = EMGetFocus( )
EMGetObjectiveStigmation( ObStigX, ObStigY )
//EMGetBrightness(Brightness)
Result( "Focus : " + focus + "\n" )  //focus is 1000 times bigger than the real value
Result( "Obj. Stig : " + ObStigX + "/"+ ObStigY + "\n" )
EMWaitUntilReady( )
// read stage position (x,y,z)
Result( "\n\n" )
Result( "Current Stage Positions: \n" )
number stageX, stageY, stageZ
stageX = EMGetStageX()
stageY = EMGetStageY()
stageZ = EMGetStageZ()
Result( "3viewStageX: " + stageX + "\n" )
Result( "3viewStageY: " + stageY + "\n" )
Result( "3viewStageZ: " + stageZ + "\n" )
EMWaitUntilReady( )
//read current beam shift value
number x_beam,y_beam
EMGetBeamShift(x_beam,y_beam)
Result( "current beam shift " +x_beam +"and y" + y_beam + "\n" )
//set beam energy value to 1.4 keV
number BeamEnergy1 = 1400  //notice the unit here is volt
EMSetBeamEnergy(BeamEnergy1)
EMWaitUntilReady( )
//acquire image at 1.4 keV
```

```
EMSetBeamBlanked(1)  //1 means beam blank
Sleep(SCOPE_DELAY)
EMSetBeamBlanked(0) //0 means beam unblank
//image img+n
image img14 :=integerimage("Imaging at" +"1.4keV"+n, 2,0,2000,2000)
EMSetBeamBlanked(1)  //1 means beam blank
Sleep(SCOPE_DELAY)
EMSetBeamBlanked(0) //0 means beam unblank
DSAcquireData(img14,1,4,0,0)
showImage(img14)
Result( "acquire image at 1.0kev DONE \n" )
EMWaitUntilReady( )
//save image at 1.4 keV
//Image fImage = GetFrontImage()
SaveAsGatan(img14, "E:\\11142017\\"+ n + "lowKeV")
Result( "IMAGE SAVE 1.4KEV done \n" )
EMWaitUntilReady( )
Result( "debug1" )
// move stage position (x,y,z)
Result( "\n\n" )
Result( "moved Stage Positions: \n" )
//number stageX1 = stageX + delta_x
//number stageY1 = stageY + delta_y
number stageZ1 = stageZ
EMSetStageX(NewStageX)
EMSetStageY(NewStageY)
EMSetStageZ(stageZ1)
Result( "3viewStageX1: " + NewStageX + "\n" )
Result( "3viewStageY1: " + NewStageY+ "\n" )
Result( "3viewStageZ1: " + stageZ1 + "\n" )
EMWaitUntilReady( )
//set beam energy value to 1.4 keV
number BeamEnergy3 = 2200
EMSetBeamEnergy(BeamEnergy3)
EMWaitUntilReady( )
 //set new mag
//number magindex = EMGetMagIndex()
//EmSetMagIndex (magindex + 1)
EMWaitUntilReady()
//number Mag = EMGetMagnification( )
//number Newmag = Mag + delta_mag
EMSetMagnification(NewMag)
EMWaitUntilReady( )
number Newmagv = EMGetMagnification( )
Result( "changed magnification: " + newMagv + "\n" )
//set new focus
```

```
//number oldfocus, newfocus
//oldfocus = EMGetFocus()
//newfocus = oldfocus + delta_focus
EMSetFocus(NewFocus)
EMWaitUntilReady( )
//AFS_Run()
//set new objstigx_y
//number newObStigX, newObStigY
number NObStigX, NObstigY
//newObStigX = ObStigX + delta_stigx
//newObStigY = ObStigY + delta_stigy
EMSetObjectiveStigmation( newObStigX, newObStigY )
EMGetObjectiveStigmation( NObStigX, NObStigY )
Result( "changed stigmation: " + NobStigX + "/" + NObStigY +"\n" )
EMSetBeamBlanked(1)  //1 means beam blank
Sleep(SCOPE_DELAY)
EMSetBeamBlanked(0) //0 means beam unblank
//EMSetObjectiveStigmation( newObStigX, newObStigY )
//read current beam shift value
number x_beam1,y_beam1
EMGetBeamShift(x_beam1,y_beam1)
Result( "current beam shift2 " +x_beam1 +"and y" + y_beam1 + "\n" )
//acquire image at 1.4 keV
image img34 :=integerimage("Imaging at" +"2.2keV"+n, 2,0,2000,2000)
EMSetBeamBlanked(1)  //1 means beam blank
Sleep(SCOPE_DELAY)
EMSetBeamBlanked(0) //0 means beam unblank
DSAcquireData(img34,1,2,0,0)
showImage(img34)
//save image at 3.4 keV
//Image fImage1 = GetFrontImage()
SaveAsGatan(img34, "E:\\11142017\\" + n + "highKeV")
//raise the sample up
number deltaZ = 0.05 //um
number stageZnew = stageZ + deltaZ
EMSetStageZ (stageZnew)
Result( "3viewStageZmoved: " + stageZnew+ "\n" )
EMSetBeamBlanked(1)  //1 means beam blank
Sleep(SCOPE_DELAY)
EMSetBeamBlanked(0) //0 means beam unblank
//cut the sample and blank the beam in this process
Number StrokeUp = 64 + 32 + 16 + 8 + 4 + 2*0 + 1*0
Number StrokeDown = 64 + 32 + 16 + 8 + 4 + 2*1 + 1*1
EMSetBeamBlanked(1)  //1 means beam blank
MicrotomeStage_Near()
DSSetDigitalOutput(StrokeUp)
```

```
MicrotomeStage_cut(1)
MicrotomeStage_Retract(1)
DSSetDigitalOutput(StrokeDown)
MicrotomeStage_Clear()
Sleep(SCOPE_DELAY)
EMSetBeamBlanked(0) //0 means beam unblank
Result( "cut process skipped " + "\n" )
//set beam energy value back to 1.0 keV
number BeamEnergy4 = 1400
EMSetBeamEnergy(BeamEnergy4)
// move stage position back (x,y,z)
Result( "\n\n" )
Result( "moved Stage Positions: \n" )
EMSetStageX(stageX)
EMSetStageY(stageY)
EMSetStageZ(stageZnew)
Result( "3viewStageX_old_value: " + stageX + "\n" )
Result( "3viewStageY_old_value: " + stageY + "\n" )
Result( "3viewStageZ_old_value: " + stageZnew + "\n" )
//set Magnification back
EMSetMagnification(Mag)
//set ObstigX and Y back
EMSetObjectiveStigmation(ObStigX, ObStigY )
//set focus back
EMSetFocus(focus)
//AFS_Run()
}
//turn off Beam energy when done
EMSetBeamBlanked(1)  //1 means beam blank
EMSetHighTensionOnOff(0)  // '1/0' = 'on/off'
```

Supplementary References

1. Ritchie N. W. M. Spectrum simulation in DTSA-II. *Microsc Microanal* **15**, 14 (2009).
